# Droplet-based microfluidic analysis and screening of single plant cells

**DOI:** 10.1371/journal.pone.0196810

**Published:** 2018-05-03

**Authors:** Ziyi Yu, Christian R. Boehm, Julian M. Hibberd, Chris Abell, Jim Haseloff, Steven J. Burgess, Ivan Reyna-Llorens

**Affiliations:** 1 Department of Chemistry, University of Cambridge, Cambridge, United Kingdom; 2 Department of Plant Sciences, University of Cambridge, Cambridge, United Kingdom; The Ohio State University, UNITED STATES

## Abstract

Droplet-based microfluidics has been used to facilitate high-throughput analysis of individual prokaryote and mammalian cells. However, there is a scarcity of similar workflows applicable to rapid phenotyping of plant systems where phenotyping analyses typically are time-consuming and low-throughput. We report on-chip encapsulation and analysis of protoplasts isolated from the emergent plant model *Marchantia polymorpha* at processing rates of >100,000 cells per hour. We use our microfluidic system to quantify the stochastic properties of a heat-inducible promoter across a population of transgenic protoplasts to demonstrate its potential for assessing gene expression activity in response to environmental conditions. We further demonstrate on-chip sorting of droplets containing YFP-expressing protoplasts from wild type cells using dielectrophoresis force. This work opens the door to droplet-based microfluidic analysis of plant cells for applications ranging from high-throughput characterisation of DNA parts to single-cell genomics to selection of rare plant phenotypes.

## Introduction

In light of recent advances in DNA synthesis and construct assembly, phenotyping of genetic circuits is on track to becoming limiting to the rate of scientific progress. This is particularly true for plant sciences, where the time required for generation of transgenic organisms ranges from months to years. Protoplasts; individual cells whose wall has been removed through mechanical or enzymatic means, offer an alternative to analysis of plant tissues and open up the possibility of high-throughput phenotyping of single cells [1]. Introduction of DNA into protoplasts by electroporation [2–7], PEG-based transfection [8, 9], or particle bombardment [10] has proven a valuable approach to transient and stable transformation of nuclear and organellular genomes, in particular for plants not amenable to Agrobacterium-mediated transgene delivery. Protoplasts have furthermore been used to overcome barriers of sexual incompatibility in generating hybrid plants with novel properties [11]. Following transformation or somatic hybridization, whole plants can be regenerated from individual protoplasts through tissue culture [12]. In addition, protoplasts have become recognized as convenient experimental systems for studying aspects of plant cell ultrastructure, genetics, and physiology [13]. However, to date protoplasts have been extracted and analysed in bulk, limiting their use.

Recently, droplet-based microfluidics has gained increasing popularity as a platform for high-throughput culture, manipulation, sorting, and analysis of up to millions of individual cells under diverse conditions [14–18]. This approach is based on pico- to nanolitre-volume aqueous microdroplets which spatially separate individual cells from one another during processing. To date, droplet-based microfluidics has primarily been applied to bacteria [19–22], unicellular eukaryotes [22–24], and non-adhesive mammalian cells [25, 26]. The prospect of utilizing this platform for characterization and screening of individual plant protoplasts is highly attractive: high-throughput screening of whole plants is substantially limited by their slow growth and size. By contrast, millions of plant protoplasts may be processed in a matter of hours using droplet-based microfluidics, which may prepare regeneration of only pre-selected protoplasts into whole plants.

Microfluidic devices have been applied for the collection and lysis [27], culture [28], chemically-induced fusion [29], electrofusion [30], regeneration [31], and developmental characterization [32] of plant protoplasts. However, platforms for the high-throughput characterization or sorting of individual plant protoplast based on their level of gene expression have been limited to date. One group has explored this approach and used optical tweezers to displace non-encapsulated plant protoplasts in a microfluidic chip, but has not demonstrated successful sorting [33]. While fluorescence-activated cell sorting (FACS) has been applied to sorting of plant cells [34–36], FACS is relatively expensive and not available for many laboratories. Moreover, debris generated during enzymatic treatment of plant tissue has been found to clog the instrument [35]. Taken together with the fragility of plant protoplasts [36], instrument clogging markedly compromises sample injection speed, lowering the rate of events analysed per second. In addition to alleviating these issues, droplet based microfluidics allows the compartmentalization of single cells, thus opening the possibility of rapid prototyping of novel biochemical pathways [22, 37–39].

In this paper, we demonstrate high-throughput characterization and sorting of plant protoplasts encapsulated individually in aqueous microdroplets, based on the genetic expression of a fluorescent reporter protein. We use protoplasts derived from the model plant *Marchantia polymorpha* [40], which combines a simple genomic structure [41, 42] with ease of handling [43] and robustness of regeneration in absence of supplemented plant hormones [44]. We enzymatically isolate *M*. *polymorpha* protoplasts from adult thalli, and encapsulate them via a flow-focusing microfluidic device. An optical detection setup integrated into the microfluidic channel allows high-throughput quantification of chlorophyll autofluorescence or promoter-controlled YFP fluorescence emitted by individual encapsulated protoplasts. We demonstrate how this droplet-based microfluidic system can be used to rapidly measure the stochastic properties of an inducible plant promoter over a population of individual plant protoplasts. We furthermore show this system is capable of automated sorting of individual encapsulated protoplasts based on their YFP fluorescence intensity. Facilitating high-throughput screening and enrichment of plant protoplasts based on expression of a fluorescent reporter gene, our microfluidic system streamlines the identification and isolation of desired genetic events in plant biology research and modern biotechnology.

## Materials and methods

### Chemicals, buffers, and media

Unless noted otherwise, chemicals used were obtained from Sigma Aldrich (Haverhill, UK) or Fischer Scientific (Loughborough, UK). DNA primers and Driselase from Basidiomycetes sp. (D8037) were obtained from Sigma Aldrich (Haverhill, UK). Standard molecular biology buffers and media were prepared as described in by Sambrook and Russell [45].

### Microfluidic device fabrication

The microfluidic device was fabricated via soft lithography by pouring poly(dimethylsiloxane) (PDMS) along with crosslinker (Sylgard 184 elastomer kit, Dow Corning, Midland, MI, USA; pre-polymer: crosslinker = 10: 1) onto a silicon wafer patterned with SU-8 photoresist [46, 47]. The mixture was degassed in a vacuum dessicator and baked at 75 °C overnight. The devices were peeled from the moulds and holes punched for inlets and outlets using a 1 mm diameter biopsy punch. The channel surface of PDMS was activated using oxygen plasma and attached to a glass slide. To ensure permanent bonding, the complete device was baked overnight at 110 °C. The inner surface of the microchannels was rendered hydrophobic by flowing trifluorooctylethoxysilane through the channels, and the device was baked at 110 °C for 2 h. Electrodes were incorporated into microfluidic chips by inserting a low-melting point indium alloy wire into a punched hole, and melting over a hot plate. Electrical wires were stripped at the end and inserted into the molten indium alloy (see also dx.doi.org/10.17504/protocols.io.ftybnpw).

### Binary vector construction

Binary vectors pCRB mpt0 (see Genbank accession No. MF939095) and pCRB PMpHSP17.8 (see Genbank accession No. MF939096) were based on pGreenII [48], and constructed by means of isothermal assembly [49]. To confer hygromycin resistance to transgenic *M*. *polymorpha*, both binary vectors contained a hygromycin phosphotransferase gene [50] expressed under control of the strong constitutive MpEF1α promoter [51]. pCRB further contained an mVenus yellow fluorescent reporter gene [52] under control of PMpEF1α. pCRB PMpHSP17.8 contained an mVenus gene under control of the heat-inducible MpHSP17.8 promoter [53].

### Transformation of *A*. *tumefaciens*

50 μL aliquots of electrocompetent *A*. *tumefaciens* GV3101(pMP90) cells containing the pSoup helper plasmid were thawed on ice, mixed with 50–100 ng of DNA at the bottom of a pre-chilled 2 mm gap electroporation cuvette (VWR, Radnor, PA, USA), and kept on ice for 15 min. Electroporation was carried out using an E. coli Pulser Transformation Apparatus (Bio-Rad, Hercules, CA, USA) according to the manufacturer’s instructions at 2.50 kV, 5 ms pulse length, and 400 Ω default resistance. 1 mL of liquid SOC medium pre-warmed to 28 °C was then immediately added to each cuvette, and the cells transferred to 15 mL Falcon tubes for recovery over 2–3 h at 28 °C under shaking (ca. 120 rpm). 250 μL of cells were then spread onto LB 1.2%(w/v) agar plates containing 25 μg/mL gentamicin, 5 μg/mL tetracycline, 50 μg/mL rifampicin, and 50 μg/mL kanamycin. Colonies became visible on the agar plates after approximately 2 days of incubation at 28m°C.

### Plant materials and growth conditions

*M*. *polymorpha* Cam-strain plants were grown on B5 medium supplemented with 1.6 g/L vitamins (1/2 B5Vit; G0210, Melford, Ipswich, UK) containing 1.2%(w/v) agar, under continuous white light.

#### Surface sterilization and germination of *M*. *polymorpha* spores

*M*. *polymorpha* sporangia (2 per nuclear transformation to be attempted) were crushed with a polypropylene cell spreader until only small fragments (< 5 mm in diameter) remained visible. Sterile dH_2_O (1 mL per nuclear transformation) was added, and the tube vortexed vigorously for 30 sec. The crushing and vortexing steps were repeated, the suspension passed through a Falcon 40 μm cell strainer (Corning, Wiesbaden, Germany) to remove plant debris, and 500 μL aliquots of the filtrate transferred into 1.5 mL Eppendorf tubes. Spores were spun down at 13,000 rpm for 1 min, and the supernatant removed without disturbing the pellet. Each pellet was then resuspended into 1 mL of a sterilizing solution prepared by dissolving 1 Milton Mini Sterilizing Tablet (Procter & Gamble, Cincinnati, OH, USA) in 25 mL of sterile dH_2_O. The tubes were shaken at room temperature for 20 min at 200 rpm. Surface-sterilized spores were then pelleted by centrifugation as above and washed by 1 mL of sterile dH_2_O. The spore content of each tube was resuspended in 100 μL of sterile dH_2_O and spread on two 1/2 B5Vit 1.2%(w/v) agar plates. The plates were sealed and kept inverted under white fluorescent light at 23 °C as described above. Small thalli were visible under a stereomicroscope after approximately 1 week.

### Nuclear transformation of *M*. *polymorpha* sporelings

2–3 colonies of *A*. *tumefaciens* GV3101 (pMP90,pSoup) carrying a binary plasmid of interest were used to inoculate 4 mL of selective LB medium supplemented by 100 μM acetosyringone, and the culture incubated overnight at 28 °C under shaking (ca. 120 rpm). 1 mL of the overnight culture was used to inoculate 4 mL of selective 1/2 B5Vit medium supplemented by 100 μM acetosyringone, 0.1%(w/v) casamino acids, 0.03%(w/v) glutamine, and 2%(w/v) sucrose (1/2 B5VitAcSuc). The diluted culture was incubated at 28 °C for 4 h under shaking (ca. 120 rpm). Germinating spores of *M*. *polymorpha* on day 6 after surface sterilization were harvested by adding 2 mL of 1/2 B5VitSuc (equals 1/2 B5VitAcSuc without acetosyringone) to each plate, resuspending germinating spores in the liquid, through scraping them off the agar using a polypropylene cell spreader, and transferring the suspension to a 50 mL Falcon tube using a pipette. For each transformation, a suspension of germinating spores corresponding to the content of 2 agar plates (i.e. 2 sporangia) was diluted into 50 mL of 1/2 B5VitAcSuc in a baffled 250 mL Erlenmeyer shaking flask. Following addition of 1 mL of transgenic *A*. *tumefaciens* GV3101 (pMP90,pSoup), subcultured in 1/2 B5VitAcSuc as described above, each flask was shaken at 150 rpm for 48 h under white fluorescent light at 23 °C. After co-cultivation, spores were rescued by passing the suspension through a Falcon 40 μm cell strainer (Corning, Wiesbaden, Germany). Collected spores were washed by 200 mL of 100 μg/mL cefotaxime in sterile dH_2_O to remove *A*. *tumefaciens*, and spread on 1/2 B5VitAcSuc 1.2%(w/v) agar plates containing 100 μg/mL cefotaxime and 20 μg/mL hygromycin. The spore content of a single shaking flask was distributed to 3–4 agar plates after collection and washing. Transgenic thalli were observed after 1–2 weeks under white fluorescent light at 2 3°C.

### Protoplast preparation

Protoplasts were isolated from *M*. *polymorpha* thalli as previously described [40], with modifications: thalli were vacuum-infiltrated by 1/2 B5 containing 2%(w/v) Driselase and 6%(w/v) Mannitol for 10 min in a glass beaker, and subsequently incubated in the dark at room temperature for 5 h. The beaker was then gently swirled for 30 sec to aid protoplast release, and the protoplast-containing suspension passed through a Falcon 40 μm cell strainer to remove debris. Protoplasts were isolated from *A*. *thaliana* as previously described [54].

### Protoplast encapsulation in microfluidic droplets

Protoplasts in the aqueous phase were encapsulated into droplets using a flow-focusing microfluidic device: the protoplast suspension was loaded into a 500 μL Hamilton Gas-tight syringe (Hamilton Robotics, Reno, NV, USA). The fluorinated oil used as the continuous phase (3M Novec 7500 Engineered Fluid with 2.5% PicoSurf 1 surfactant, Sphere Fluidics, Cambridge, UK) was loaded in another syringe and both syringes were connected to the respective inlets of the flow-focusing device (nozzle dimensions: 40 μm x 40 μm x 50 μm) with fine bore polyethylene tubing (ID = 0.38 mm, OD = 1.09 mm, Smiths Medical International, Luton, UK). Using syringe pumps (PHD 2000 Infusion, Harvard Apparatus, Holliston, MA), the two solutions were injected simultaneously in the device. The oil phase was injected at a rate of 500 μL/h and the aqueous phase at a rate of 300 μL/h. The generated droplets were collected, through tubing connected to the outlet, into a syringe.

### Bright-field and fluorescence microscopy

Microdroplet formation was monitored using a Phantom V72 fast camera (Vision Research, Wayne, NJ, USA) mounted on an inverted microscope (IX71, Olympus, Tokyo, Japan). Videos of the encapsulation procedure were captured using the supplied Phantom software. Protoplasts encapsulated in microdroplets were imaged using an inverted microscope (IX71, Olympus, Tokyo, Japan). Chlorophyll fluorescence was excited at 642–682 nm and collected at 603.5–678.5 nm. YFP fluorescence was excited at 488–512 nm and collected at 528.5–555.5 nm.

### On-chip fluorescence measurements and sorting of encapsulated protoplasts

To measure protoplasts fluorescence in each microdroplet, a fixed 491 nm wavelength laser (Cobolt AB, Solna, Sweden) was shaped into a light sheet at 50 mV. The laser was focused through an UPlanFL N 20x microscope objective and directed to the microfluidic chip placed on the stage of an inverted microscope (IX71, Olympus, Tokyo, Japan). Fluorescence detection was carried out by a custom multi-part optical instrument. All filters used in this setup were purchased from Semrock (Rochester, NY, USA). Notably, emitted fluorescence was filtered through a 495 nm long-pass filter to eliminate the 491 nm excitation band. Fluorescence was recorded by a PMT (H8249, Hamamatsu Photonics, Shizuoka, Japan), and the data collected was sent to a computer through a DAQ data acquisition card (National Instruments, Austin, TX, USA). The program LabVIEW (National Instruments, Austin, TX, USA) was used to monitor and analyse the data. A microfluidic device was used for sorting YFP-expressing protoplasts in microdroplets: as the microdroplets passed through the objective field of view, they were illuminated by a 491 nm laser. Emitted fluorescence filtered through a 528.5–555.5 nm YFP band-pass filter was collected by the PMT and triggered a pulse generator connected to a high-voltage power supply. The resulting electrode pulse (200 V) deformed YFP-positive microdroplets and targeted them to a small ‘positive’ channel for collection. If the microdroplet was empty or contained protoplast lacking detectable YFP, the PMT sent no signal and the microdroplet passed through the larger ‘negative’ channel.

### Data

Extended protocols can be found at dx.doi.org/10.17504/protocols.io.ftnbnme and dx.doi.org/10.17504/protocols.io.ftybnpw.

## Results and discussion

**Fig 1:**
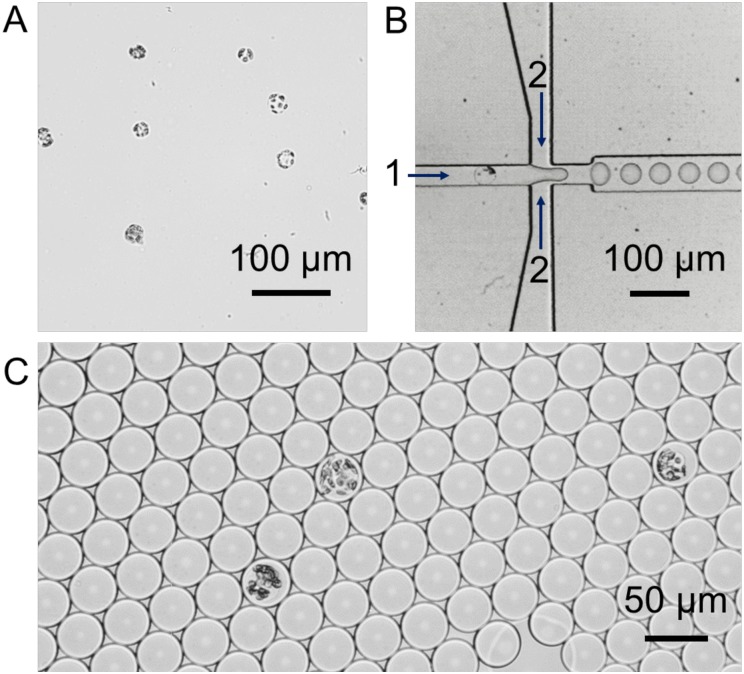
Encapsulation of *M*. *polymomrpha* protoplasts. (A) Bright field micrograph of *M*. *polymorpha* protoplasts isolated from mature thalli. (B) Bright field micrograph of a flow-focusing microfluidic device for encapsulation of *M*. *polymorpha* protoplasts in water-in-oil microdroplets. (C) Bright field micrograph of individual *M*. *polymorpha* protoplast encapsulated in microdroplets.

Isolated *M*. *polymorpha* protoplasts were encapsulated in microdroplets on a flow-focusing microfluidic device based on [18] (Fig 1A). The aqueous protoplasts suspension flowed perpendicularly to two streams of fluorinated carrier oil containing PicoSurf1 non-ionic surfactant. The two phases intersected at the ‘flow-focusing junction’, as the oil streams enveloped the droplet that budded off from the aqueous stream (Fig 1B). The density of *M*. *polymorpha* protoplasts was adjusted to ensure microdroplets contained no more than one protoplast each (Fig 1C), which is important for accurate quantification of cellular fluorescence intensity. The same approach was also successful for encapsulation of the widely used angiosperm model *Arabidopsis thaliana* (S1 Fig).

**Fig 2:**
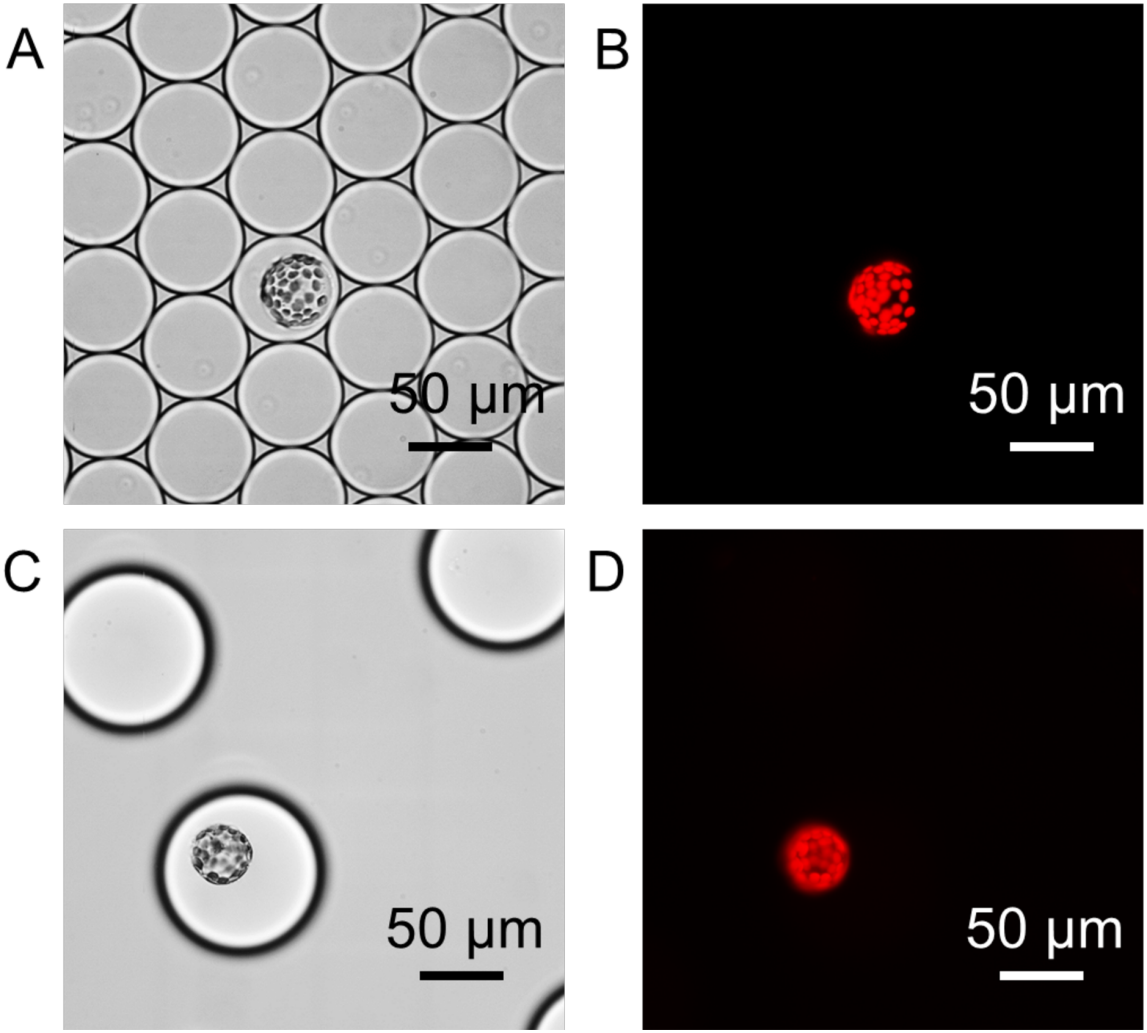
Protoplast stability after encapsulation. (A) Bright field and (B) chlorophyll fluorescence micrographs of individual *M*. *polymorpha* protoplast encapsulated in microdroplets.

**Fig 3:**
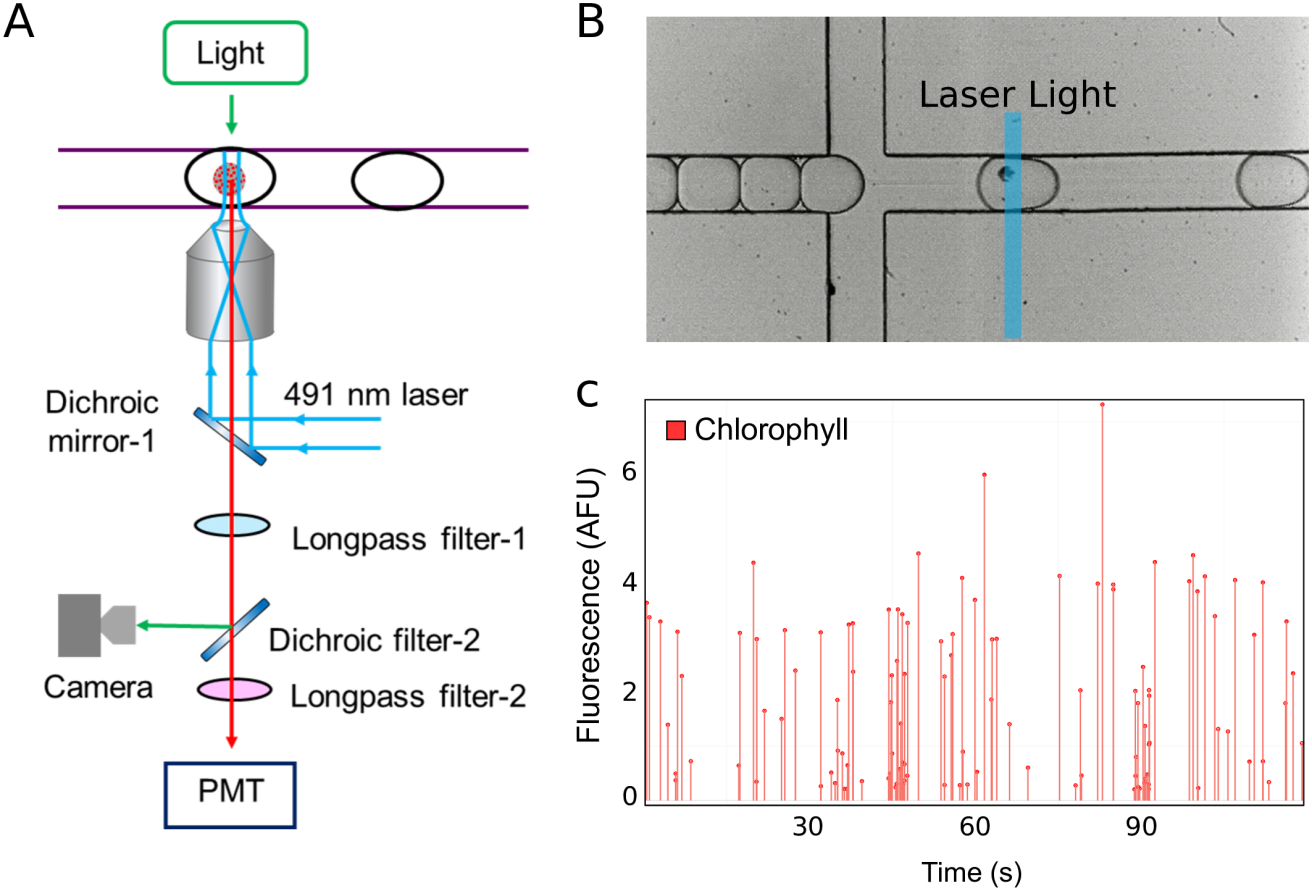
Chlorophyll fluorescence intensity of encapsulated protoplasts. (A) Experimental setup used for quantification of fluorescence intensity of encapsulated protoplasts. Long-pass filter-1: 495 nm; long-pass filter-2: 633 nm; dichroic filter-1: 495 nm; dichroic filter-2: 633 nm. (B) Bright field micrograph of an encapsulated protoplast passing through the excitation laser beam. (C) Representative PMT readout of chlorophyll fluorescence intensity recorded on chip over 120 s. Each line represents an individual encapsulated protoplast.

**Fig 4:**
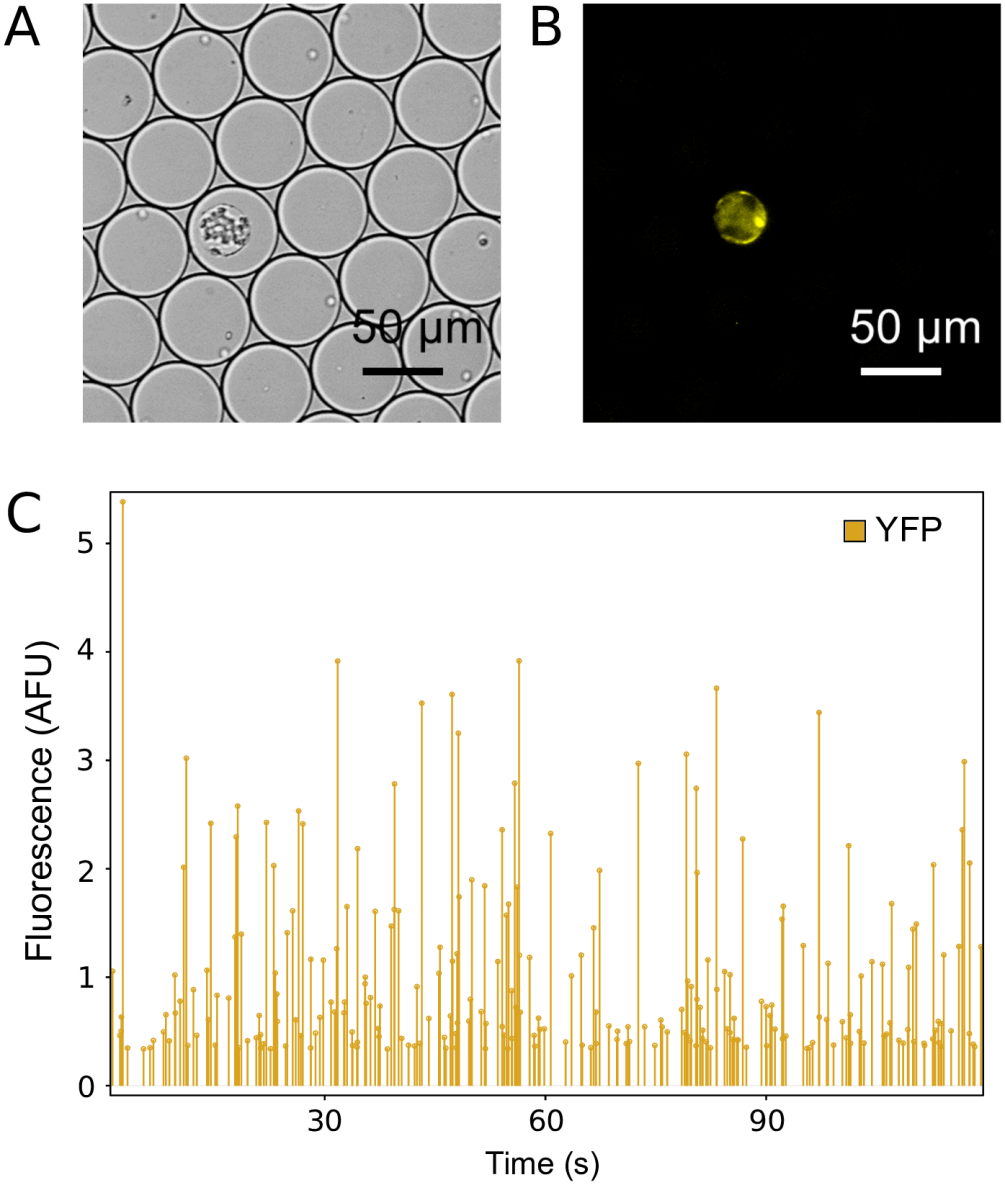
Reporter fluorescence quantification on encapsulated protoplasts. (A) Bright field and (B) mVenus fluorescence micrograph of an individual encapsulated protoplast derived from transgenic mpt0 *M*. *polymorpha* constitutively expressing mVenus. (C) Representative PMT readout of mVenus fluorescence intensity recorded on chip over 120 s. Each line represents an individual encapsulated protoplast.

While encapsulated, protoplasts remained intact over a period of at least 12 hours (Fig 2). To quantify chlorophyll autofluorescence in individual encapsulated protoplasts, an optical setup was integrated to the system (Fig 3). Each microdroplet was re-injected into a microfluidic flow channel continuously exposed to a 491 nm laser beam. Fluorescence emitted from excited protoplasts passed through a 633 nm longpass filter and the signal was collected by a photomultiplier tube (PMT; S1 Video). Using this experimental approach, the fluorescence of each protoplast was quantified for up to 115,200 individual protoplasts per hour. This observation suggests that high-throughput quantification of chlorophyll fluorescence using our microfluidic setup can be utilized for assessment of the quality of a protoplast preparation. The same experimental approach was also used for quantification of reporter protein fluorescence in individual plant cells, as illustrated by protoplasts derived from transgenic mpt0 *M*. *polymorpha* constitutively expressing mVenus [41] yellow fluorescent protein (YFP) under control of the strong constitutive MpEF1α promoter [42] (Fig 4).

As the next step, our system was applied for the analysis of the stochastic activity of an inducible promoter across a population of individual plant cells. For this purpose, transgenic PMpHSP17.8 lines of *M*. *polymorpha* were generated, which expressed mVenus yellow fluorescent protein (YFP) [52] under control the endogenous heat-responsive MpHSP17.8 promoter. It was previously shown that incubation of transgenic *M*. *polymorpha* at 37°C for 1 h induced a PMpHSP17.8-controlled targeted gene by approximately 700-fold [53].

**Fig 5:**
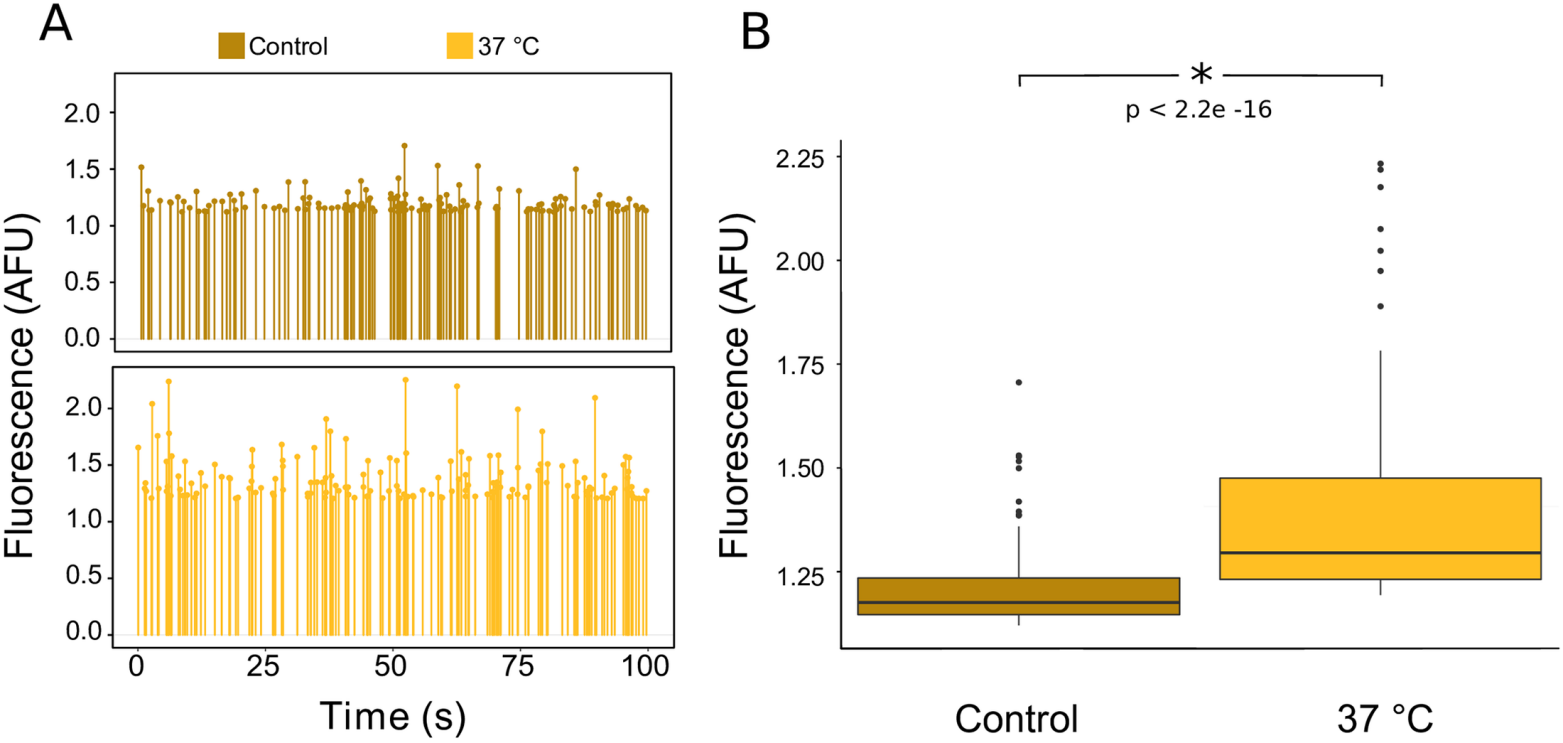
Characterization of heat-responsive induction of mVenus (YFP) in transgenic PMpHSP17.8 *M*. *polymorpha* in individual encapsulated protoplasts. Transgenic PMpHSP17.8 *M*. *polymorpha* encoding mVenus under control of the MpHSP17.8 promoter were either subjected to 4 h at room temperature (Control) or to 2 h at 37 °C followed by 2 h at room temperature (22 °C). (A) Representative PMT readout of YFP fluorescence intensity for protoplasts isolated from thalli subjected to either temperature treatment. Each line represents an individual encapsulated protoplast. (B) Boxplot of the difference in YFP fluorescence intensity between the two temperature treatments based on protoplast populations recorded on chip over 100 s each. The p value shown was calculated using unpaired t-test.

To measure the stochastic properties of this promoter, transgenic PMpHSP17.8 *M*. *polymorpha* was incubated under two different temperature conditions and isolated protoplasts from each sample for on-chip quantification of YFP fluorescence (Fig 5). *M*. *polymorpha* thalli were either subjected to (i) 2 h at 37 °C followed by 2 h at room temperature or to (ii) 4 h at room temperature (control). Protoplasts isolated from heat-shocked plants exhibited significantly higher levels of YFP activity compared to the Control (p < 2.2e-16, 95% CI [-0.2, -0.13]). This result illustrates the power of our microfluidic system to quantify stochastic properties of plant promoters as a function of environmental conditions.

**Fig 6:**
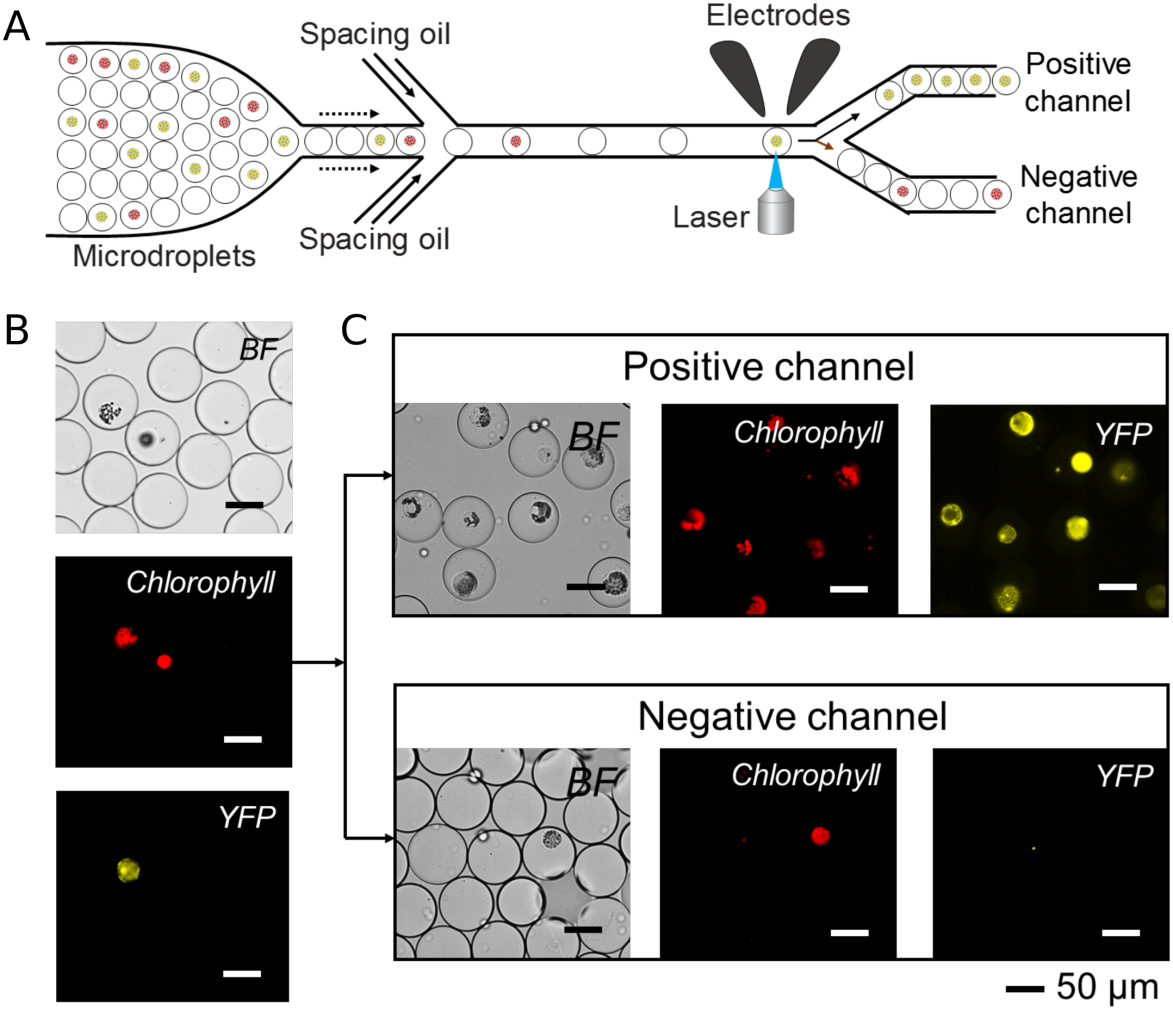
Sorting of *M*.*polymorpha* protoplasts. (A) Schematic representation of a platform for microfluidic sorting of encapsulated protoplasts. (B) Bright field and fluorescence micrographs of adjacent microdroplets containing protoplasts derived from wild-type and transgenic mpt0 *M*. *polymorpha*, respectively. (C) Bright field and fluorescence micrographs of microdroplets sorted into positive and negative channels based on their mVenus fluorescence intensity.

An even more powerful application of our microfluidic platform is sorting of individual encapsulated protoplasts based on their level of expression of a target reporter gene. This allows single plant cells to be pre-screened for downstream sequencing and/or regeneration of whole plants. For this purpose, a microdroplet-based microfluidic sorting system was developed (Fig 6A): two oil flow-focusing channels allowed the spacing between microdroplet to be controlled by flow-rate adjustment. Microdroplet sorting was implemented by a pair of electrodes generating a dielectrophoretic force applied to the microdroplet. When the electrodes were off, the microdroplets were pushed into the “negative” channel due to its lower fluidic resistance compared to the “positive” channel. Switching the electrodes on steered the individual microdroplets into the “positive” channel through dielectrophoretic force. The generation of an electrode pulse was dependent on the fluorescence intensity emitted from each microdroplet: microdroplets were steered to the “positive” channel only if they contained a protoplast expressing YFP above-threshold levels of 1.3 arbitrary fluorescence units (AFU; S2 Video). The platform was tested for using microdroplets containing protoplasts isolated from either wild type or transgenic mpt0 *M*. *polymorpha*. Protoplast from both populations were pooled together and reinjected into the sorting device (Fig 6B). Sorting successfully separated mVenus-expressing mpt0 protoplasts from ruptured cells, wild type protoplasts and empty droplets. Fig 6C shows representative microdroplets collected through the “positive” and “negative” channels. All the microdroplets collected through the “positive” channel contained While microdroplets collected through the “positive” channel contained mVenus-expressing mpt0 protoplasts. Among the “negative” channel we observed wild type protoplasts and a considerable number of empty droplets (Fig 6C and S2 Fig). This result showed our microfluidic platform capable of high-throughput selection of desired events across large populations of genetically diverse individual plant cells.

While microdroplet based microfluidics has previously been applied to unicellular photosynthetic eukaryotes [21] the work presented here allows for rapid screening of synthetic circuits designed for operation in multicellular plants. Although further work will be required to assess the capacity for regeneration of whole plants from individual sorted protoplasts, this work provides a fundament for the development of techniques reducing the time and cost involved in the screening of transgenic plants, by alleviating the need to maintain and cultivate large quantities of callus prior to selection.

## Conclusions

We have developed a droplet-based microfluidic platform for high-throughput characterization of plant protoplasts. Our device is capable of quantifying chlorophyll and GFP fluorescence of individual encapsulated cells as a function of genetic circuit activity or in response to environmental stimuli. This workflow allows collection of substantial amounts of biological information from comparatively little plant material. We expect our droplet-based microfluidic platform to be applied for screening of synthetic genetic circuits as well as of mutagenized and enhancer trap lines of a variety of plant species. In the future, we envision a microfluidic workflow composed of on-chip transformation, characterization, and fluorescence-based selection of individual plant cells in preparation of targeted regeneration into whole plants. Combined with libraries of guide RNAs and gene editing tools such as CRISPR-Cas9 nuclease, this workflow promises to greatly accelerate academic and industrial research in modern plant biotechnology.

## Supporting information

S1 Fig Encapsulation of *A*. *thaliana* protoplasts. A) Bright field and (B) chlorophyll fluorescence micrographs of individual *A*. *thaliana* leaf protoplasts encapsulated in microdroplets. (C) Representative photomultiplier tube (PMT) readout of chlorophyll fluorescence intensity represented as arbitrary fluorescent units (AFU) recorded over 17.5 s. Each line represents an individual encapsulated protoplast.
(TIF)

S2 Fig Sorting of *M*. *polymorpha* protoplasts. Bright field and fluorescence micrographs of microdroplets sorted into positive and negative channels based on their mVenus fluorescence intensity. Scale bars; 50 μm.
(PDF)

S1 Video Encapsulation of *Arabidopsis thaliana* protoplasts. (ZIP)

S2 Video Sorting of *M*. *polymorpha*protoplasts expressing YFP. (ZIP)
