# Supplementary figures and images for: Droplet-based microfluidic analysis and screening of single plant cells
Source: PLoS One. 2018 May 3;13(5):e0196810. doi: 10.1371/journal.pone.0196810 (PMC5933695; doi:10.1371/journal.pone.0196810)

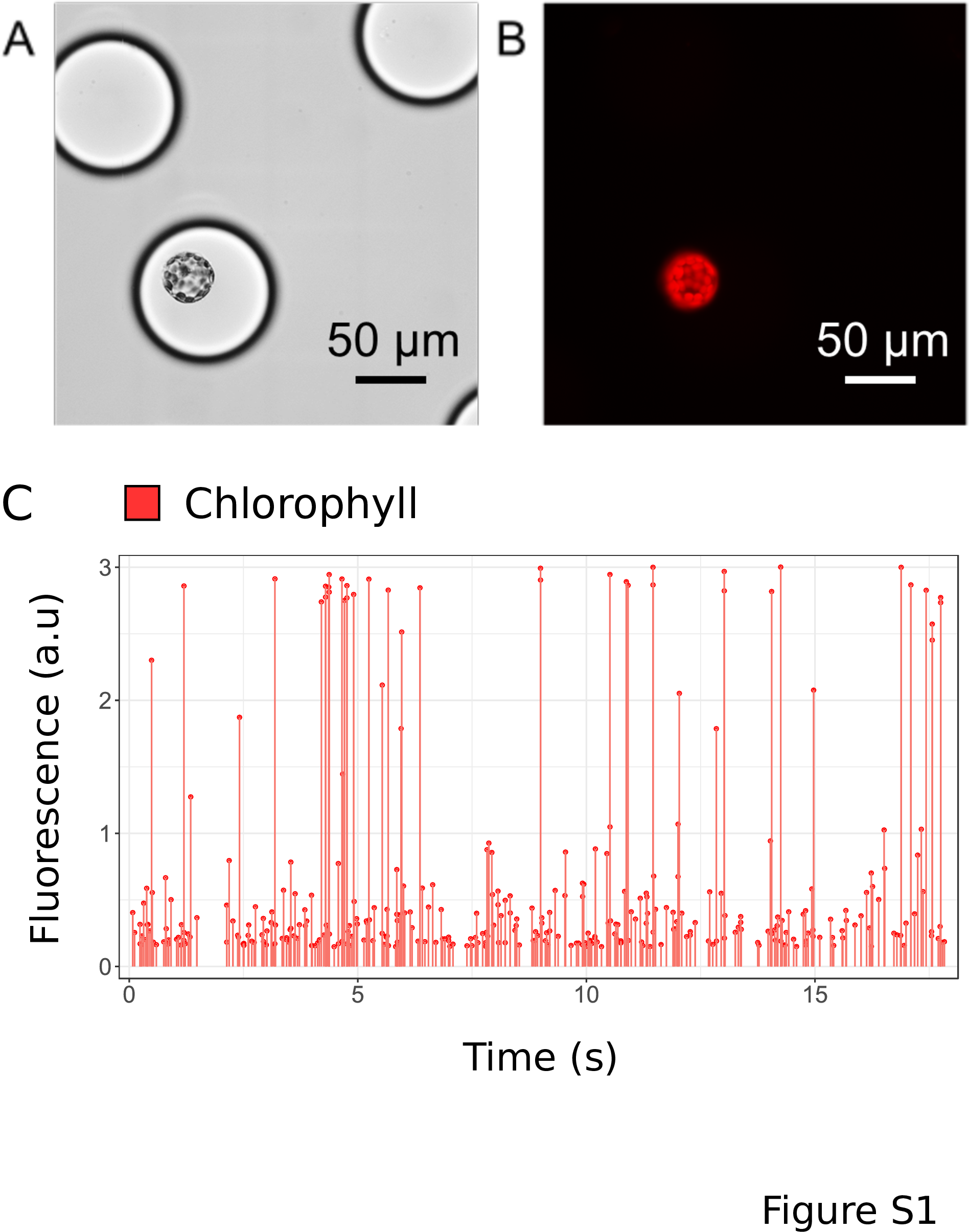

Supplement: S1 Fig — A) Bright field and (B) chlorophyll fluorescence micrographs of individual A. thaliana leaf protoplasts encapsulated in microdroplets. (C) Representative photomultiplier tube (PMT) readout of chlorophyll fluorescence intensity represented as arbitrary fluorescent units (AFU) recorded over 17.5 s. Each line represents an individual encapsulated protoplast. (TIF) [file pone.0196810.s001.tif]

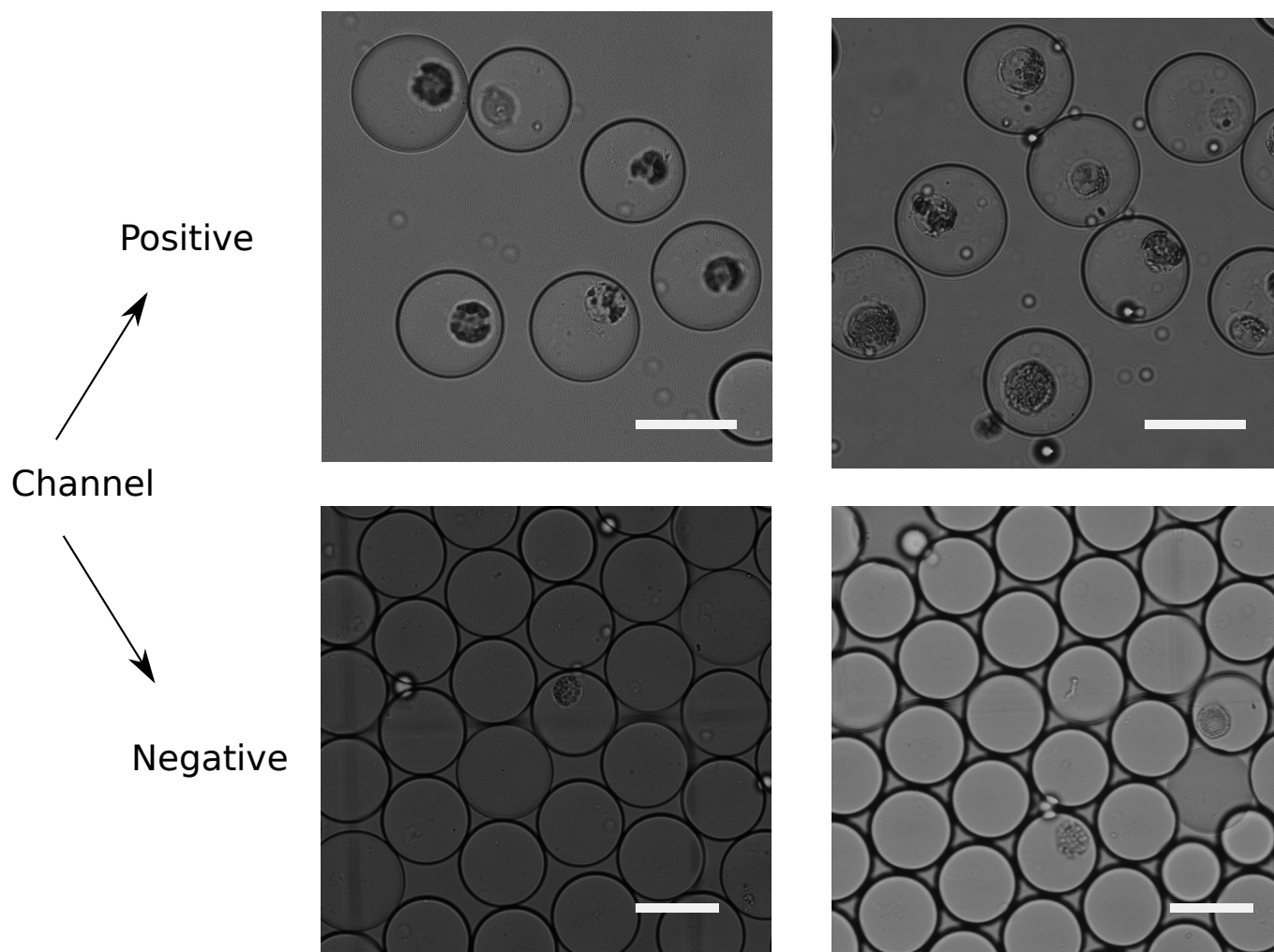

Figure S2

Supplement: S2 Fig — Bright field and fluorescence micrographs of microdroplets sorted into positive and negative channels based on their mVenus fluorescence intensity. Scale bars; 50 μm. (PDF) [file pone.0196810.s002.pdf]
